# Generation and characterization of human induced pluripotent stem cells (iPSCs) from hand osteoarthritis patient-derived fibroblasts

**DOI:** 10.1038/s41598-020-61071-6

**Published:** 2020-03-06

**Authors:** R. Castro-Viñuelas, C. Sanjurjo-Rodríguez, M. Piñeiro-Ramil, T. Hermida-Gómez, S. Rodríguez-Fernández, N. Oreiro, J. de Toro, I. Fuentes, F. J. Blanco, S. Díaz-Prado

**Affiliations:** 1 Cell Therapy and Regenerative Medicine Group, Department of Physiotherapy, Medicine and Biomedical Sciences, Faculty of Health Sciences, University of A Coruña (UDC), A Coruña, Spain; 2 Tissular Bioengineering and Cell Therapy Unit (GBTTC-CHUAC), Rheumatology Group, A Coruña, Spain; 3 Centro de Investigación Biomédica en Red (CIBER) de Bioingeniería, Biomateriales y Nanomedicina (CIBER-BBN), Madrid, Spain; 4 Institute of Biomedical Research of A Coruña (INIBIC), University Hospital Complex A Coruña (CHUAC), Galician Health Service (SERGAS), A Coruña, Spain; 5 Leeds Institute of Rheumatic and Musculoskeletal Disease, University of Leeds, Leeds, UK; 6 Centro de Investigaciones Científicas Avanzadas (CICA), Agrupación estratégica CICA-INIBIC, University of A Coruña, A Coruña, Spain

**Keywords:** Induced pluripotent stem cells, Osteoarthritis

## Abstract

Knowledge and research results about hand osteoarthritis (hOA) are limited due to the lack of samples and animal models of the disease. Here, we report the generation of two induced pluripotent stem cell (iPSC)-lines from patients with radiographic hOA. Furthermore, we wondered whether these iPSC-lines carried single nucleotide polymorphisms (SNPs) within genes that have been associated with hOA. Finally, we performed chondrogenic differentiation of the iPSCs in order to prove their usefulness as cellular models of the disease. We performed a non-integrative reprogramming of dermal fibroblasts obtained from two patients with radiographic rhizarthrosis and non-erosive hOA by introducing the transcriptional factors Oct4, Sox2, Klf4 and c-Myc using Sendai virus. After reprogramming, embryonic stem cell-like colonies emerged in culture, which fulfilled all the criteria to be considered iPSCs. Both iPSC-lines carried variants associated with hOA in the four studied genes and showed differences in their chondrogenic capacity when compared with a healthy control iPSC-line. To our knowledge this is the first time that the generation of iPSC-lines from patients with rhizarthrosis and non-erosive hOA is reported. The obtained iPSC-lines might enable us to model the disease *in vitro*, and to deeper study both the molecular and cellular mechanisms underlying hOA.

## Introduction

Osteoarthritis (OA) is a prevalent musculoskeletal disease that affects the joints, and it has a substantial effect on quality life^1–4^. Hand OA affects predominantly the carpometacarpal joint (CMCJ), followed by the interphalangeal joints (IPJs), both proximal and distal^4^. OA of the CMCJ, also known as rhizarthrosis or thumb OA, is the most common location in women over 55 years, and the severity is usually linked to handedness. Mechanical pain at the base of the thumb and the thenar eminence are the principal clinical manifestations of this pathology^5^. Erosive hand OA is another form that may involve the CMCJ of the thumb as well as IPJs, in which central erosions are found in the subcondral bone^4^.

Although both the individual and the societal burden of hand OA are well known, knowledge and research results about its underlying cellular and molecular mechanisms are limited^3^, mainly due to the dearth of tissue samples and lack of animal models of this pathology^4,6^. Cellular *in vitro* models are meaningful tools to shed light on the molecular mechanisms and pathways that are involved in hand OA. Primary chondrocytes, immortalized cell lines and mesenchymal stromal cells are commonly used as *in vitro* models of OA^6^. However, they present several limitations such as loss of their phenotype and differentiation potential after several passages or difficult harvesting, among others^7,8^.

In 2007, Takahashi and Yamanaka demonstrated that adult cells could be reprogrammed to generate induced pluripotent stem cells (iPSCs) by ectopic expression of 4 transcriptional factors: Octamer-binding transcription factor 3/4 (Oct3/4), Sex determining region Y-box 2 (Sox2), Krüpple-like factor 4 (Klf4) and c-Myc^9^. The potential of iPSCs is irrefutable because they can be used as an abundant, accessible, and autologous cell source with differentiation potential to develop human *in vitro* models while bypassing ethical concerns. In fact, several studies have been recently developed using patient-specific iPSCs^10–12^ for modeling cartilage and other aging-related diseases^13^ such as rheumatoid arthritis and knee OA^14,15^.

In the case of hand OA there is currently high controversy about how likely it is to be modelled *in vitro*, since it is a complex disease affected by several factors^16,17^. However, a strong genetic component is thought to be present in the development of hand OA, especially in women^1,18^. Additionally, Genome-Wide Association Studies (GWAS) have proposed that multiple single nucleotide polymorphisms (SNPs) in certain genes can play distinct roles in the OA pathogenesis^2,19^. Specifically, sequence variants in genes associated with growth factor signaling or genes that relate to inflammatory pathways have been shown to confer risk of OA in the hand^16,20–25^ and therefore, we think that it is very relevant to develop a model of hand OA for studying the use of these SNPs.

In this study we aimed to generate iPSC lines from patients with OA of the hand and to demonstrate the usefulness of these cells as *in vitro* cellular models for studying the pathogenesis of this disease. Furthermore, we have investigated whether the generated iPSC-lines carried sequence variants within several genes that have been suggested as implicated in the development and progression of hand OA. Finally, we evaluated the capacity of the generated iPSCs for differentiation into chondrocyte-like cells. We developed a novel differentiation protocol involving directed differentiation in micromasses.

## Results

### Isolation and characterization of human fibroblasts

We isolated cells from skin biopsies of two patients (52 and 76-years-old women respectively), henceforth named as OA patient 1 and OA patient 2, by using the explant culture technique. OA patient 1 presented radiographic non-erosive hand OA, and rhizarthrosis in the right hand. No signs of OA in knee or hip were detected radiographically, but so in lumbar column. OA patient 2 presented radiographic non-erosive hand OA, and rhizarthrosis in both hands. No signs of OA in knee or hip were detected radiographically. No radiographic information was available regarding lumbar column.

**Figure 1:**
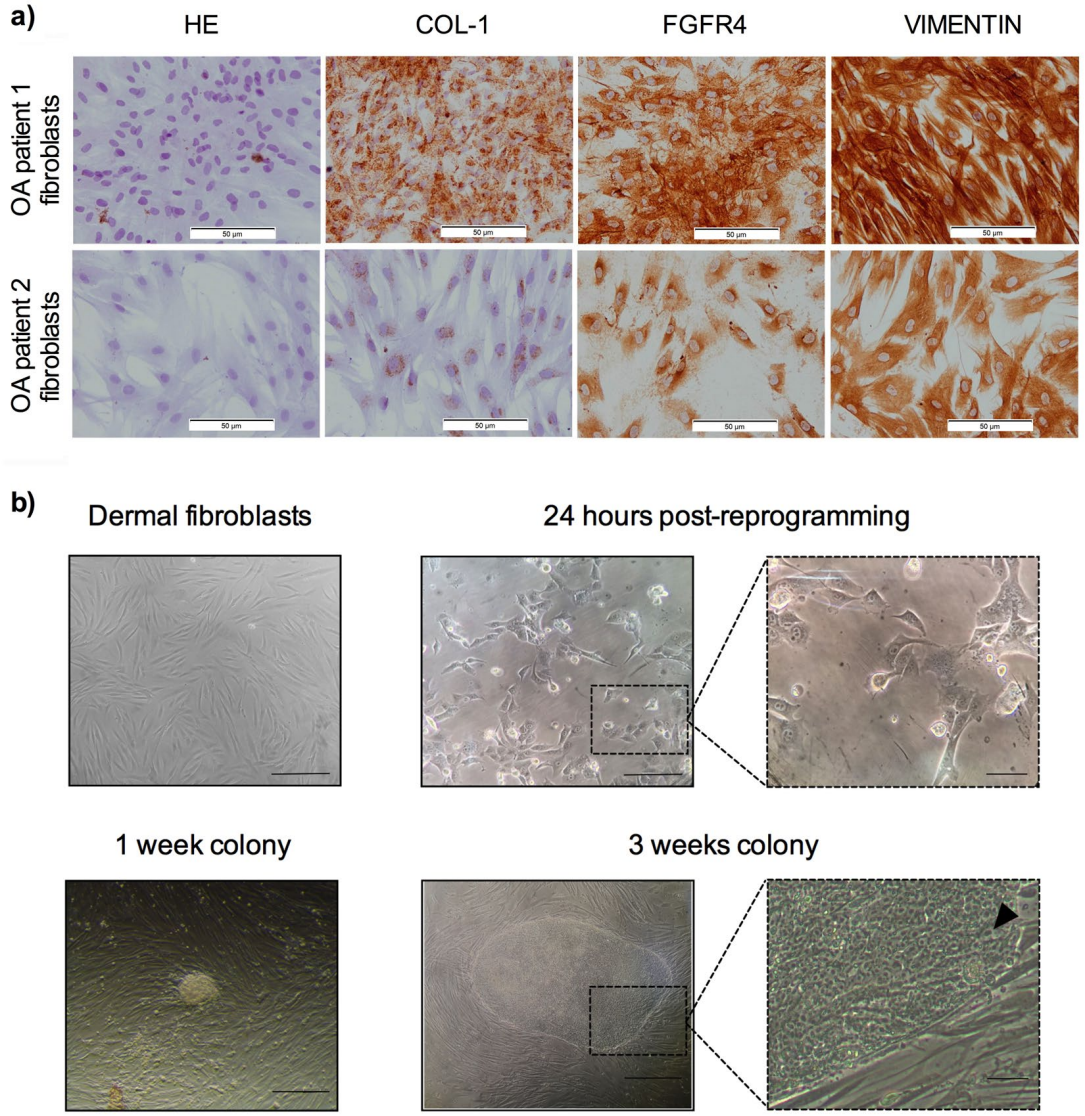
Characterization of fibroblasts and reprogramming process. (**a**) Images of hematoxylin-eosin (HE) staining, type I collagen (COL-1), acidic fibroblast growth factor receptor 4 (FGFR4) and vimentin immunostainings performed on fibroblast cultures, obtained from the OA patients 1 and 2. Scale 100 μm. (**b**) Phase contrast images taken after fibroblasts isolation (scale 200 μm) and reprogramming, showing the morphological changes occurred after transduction (scale 200 μm and 50 μm, respectively), as well as iPSC colonies morphology one (scale 200 μm) and three weeks after reprogramming (scale 200 μm and 50 μm, respectively). The black arrow points the high nucleus/cytoplasm ratio found in the iPSC-colonies.

Sufficient number of cells for analysis and reprogramming was obtained after two weeks in culture. Immunohistochemical analysis revealed that approximately 90% of cells in culture showed positive staining for the fibroblast marker proteins type I collagen, acidic fibroblast growth factor receptor 4 (FGFR4) and vimentin (Fig. 1a). Phase contrast images showed uniform cell populations with typical fibroblast-like morphology (Fig. 1b).

### Human fibroblast reprogramming gave rise to ESC-like colonies

To generate human iPSCs from OA patients minimizing the risk of genomic abnormalities, we introduced the OSKM factors using the non-integrating technology that includes modified Sendai RNA viruses. Interestingly, 24 hours after the reprogramming process, dermal fibroblasts experimented marked morphological changes, from the initial spindle-shape to a more polygonal or epithelial-like cells morphology (Fig. 1b). Twenty-two days after reprogramming colonies showing a typical human ESC-like morphology appeared in culture (Fig. 1b). Based on colonies morphology and Alkaline phosphatase (AP) activity staining, the calculated reprogramming efficiency was 0.2% for the OA patient 1 and 0.95% for the OA patient 2. Fragments of ESC-like colonies after picking were able to form new colonies (clones) onto feeder cells.

### iPSC characterization

#### iPSC colonies presented alkaline phosphatase activity

**Figure 2:**
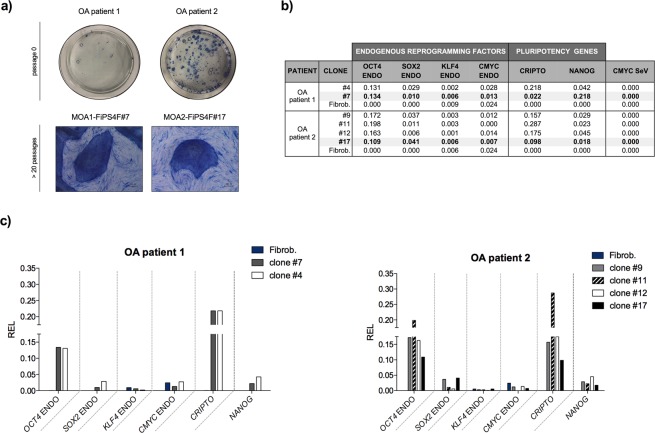
Alkaline phosphatase activity and relative expression levels of the endogenous reprogramming factors and pluripotency markers in the reprogrammed cells. (**a**) Alkaline phosphatase staining of iPSCs colonies at passage 0 and iPSCs lines MOA1-FiPS4F#7 and MOA2-FiPS4F#17 with more than 20 passages in culture. Scale bar 100 μm. (**b**) Table showing the relative expression levels (REL) of endogenous reprogramming factors (OCT4 ENDO, SOX2 ENDO, KLF4 ENDO, CMYC ENDO), pluripotency markers (CRIPTO, NANOG) and Sendai virus reprogramming factor CMYC (CMYC SeV) in the analysed clones and parental fibroblasts of OA patient 1 and OA patient 2. (**c**) Bar graphs with qRT-PCR data showing the REL of endogenous reprogramming factors and pluripotency markers.

The newly raised colonies after the reprogramming process appeared in blue when treated with AP kit, showing that they presented AP activity. The iPSC clones also stained strongly positive for AP activity, and this positivity was maintained after passaging (Fig. 2a).

#### iPSC clones express key pluripotency marker genes

According to morphological features and positive AP staining, we selected two clones (#4 and #7) of the OA patient 1, and four clones (#9, #11, #12 and #17) of the OA patient 2 to be molecularly characterized.

qRT-PCR analyses showed that all chosen clones expressed the endogenous reprograming factors (OCT4 ENDO, SOX2 ENDO, KLF4 ENDO and CMYC ENDO) as well as genes characteristic of human ESCs, including NANOG and CRIPTO. Relative expression levels (REL) for these key pluripotency genes were markedly increased in the iPSCs compared to the parental dermal fibroblasts (Fig. 2b,c), except for endogenous KLF4 and CMYC, which were slightly higher in the parental fibroblasts.

Among clones selected from the healthy donor, clone #H was the one with higher expression of both CRIPTO and NANOG. However, expression of Sendai virus cMYC (CMYC SV) was also detected. Clone #E expressed high relative expression levels of OCT4 ENDO and CRIPTO, but not NANOG. Clones #2 and #7 expressed similar levels of all genes, excepting CRIPTO and SOX2 ENDO, which were higher in clone #2. Regarding OA patient 1, both clones #4 and #7 expressed high relative expression levels of OCT4 ENDO and CRIPTO compared to those in the parental dermal fibroblasts. NANOG, SOX2 ENDO and CMYC ENDO levels were slightly higher in clone #4 than in clone #7. Finally, all clones selected from OA patient 2 expressed similar REL of the endogenous reprogramming factors and the pluripotency markers, being these levels higher than the ones observed in the native dermal fibroblasts. Clones #9 and #12 presented higher relative expression levels of CMYC ENDO when comparing with clones #11 or #17.

Due to the lower expression of CMYC ENDO, all together with high REL of the pluripotency markers CRIPTO, NANOG and OCT4 ENDO, the candidate clones selected to be further characterized were clone #7 from the OA patient 1 and clone #17 from de OA patient 2.

#### iPSC clones stained positive for pluripotency markers

**Figure 3:**
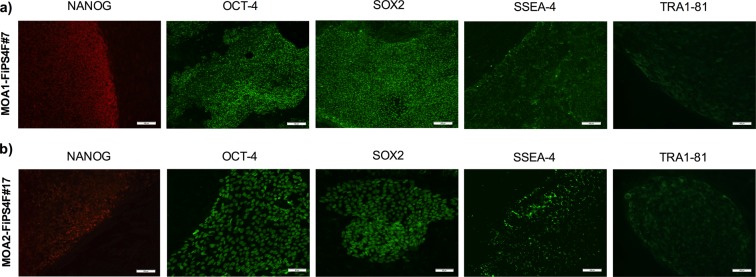
Immunofluorescence analisys of pluripotency-associated markers. (**a**) Immunofluorescence staining showing presence of pluripotency markers NANOG, OCT-4, SOX2, SSEA-4 and TRA-1-81 in the iPSC-line MOA1-FiPS4F#7. Scale 100 μm. (**b**) Immunofluorescence staining showing presence of pluripotency markers NANOG (scale 100 μm), OCT-4 (scale 50 μm), SOX2 (scale 50 μm), SSEA-4 (scale 100 μm) and TRA-1-81 (scale 100 μm) in the iPSC-line MOA2-FiPS4F#17.

The two studied clones were intracellularly stained (nucleus) for NANOG, OCT-4 and SOX2 self-renewal markers. These clones were also positive for SSEA-4 and TRA-1-81 pluripotency surface markers (Fig. 3).

#### Generated iPSCs are able to differentiate *in vitro* towards the three embryonic germ layers

**Figure 4:**
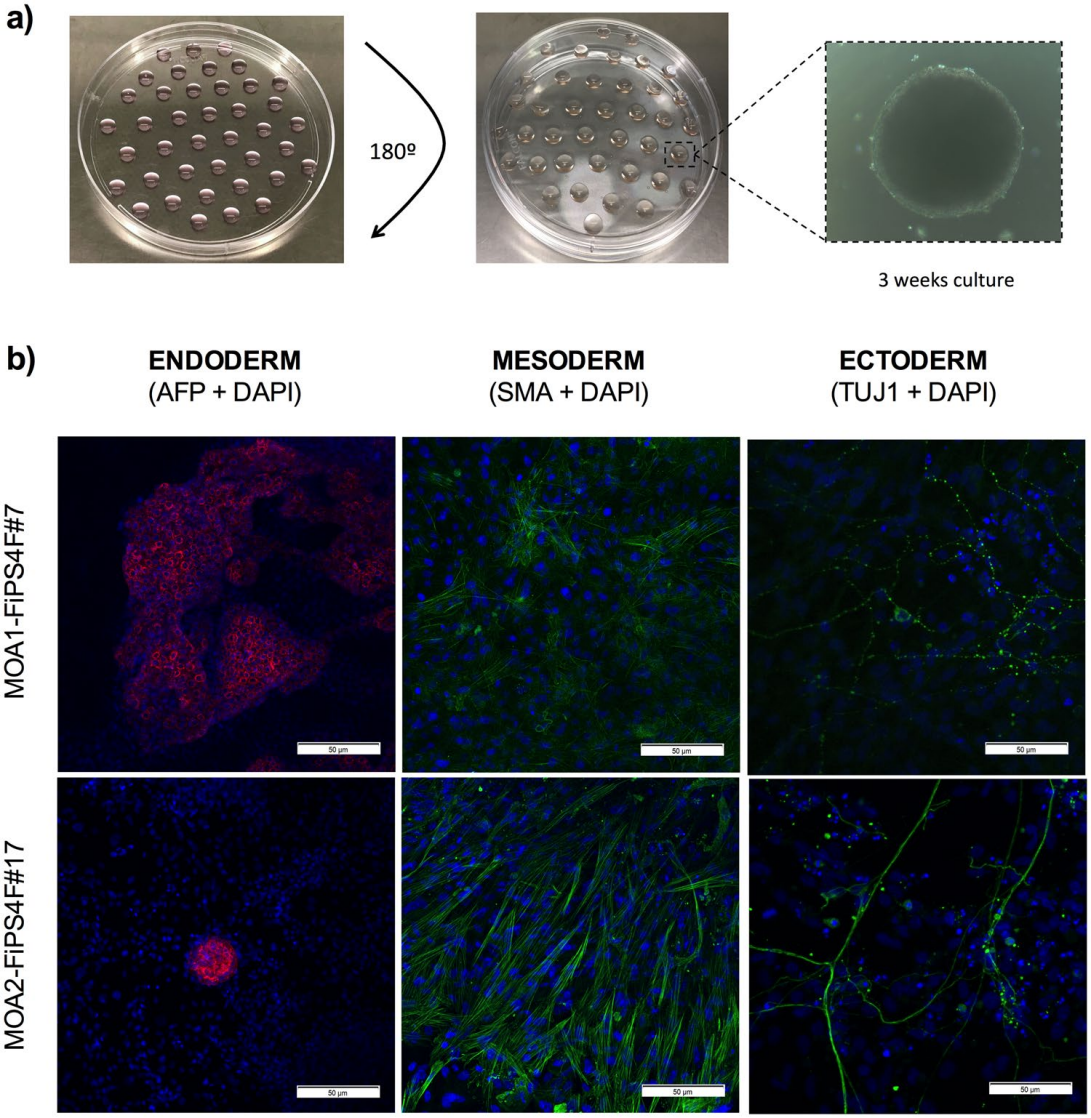
Analysis of the functional pluripotency in the reprogrammed cells. (**a**) Scheme representing the hanging drop technique used to form embryoid bodies (EBs). EBs were generated to study trilineage differentiation of the iPSC-lines. (**b**) Immunofluorescence staining showing presence of α-fetoprotein (AFP, ectoderm), α-smooth muscle actin (SMA, mesoderm) and β-III-Tubulin (TUJ1, ectoderm) in the iPSC-lines MOA1-FiPS4F#7 and MOA2-FiPS4F#17. DNA was counterstained with DAPI (scale 50 μm).

In order to check the *in vitro* pluripotency of the iPSCs, tri-lineage differentiation was performed using the embryoid body (EB) protocol. The hanging-drop technique enabled us to generate EBs within 48 hours in culture (Fig. 4a). We observed heterogeneous cell populations sprouted and grown out of the EBs after one week in culture. The differentiation potential of the generated iPSCs was confirmed through detection of immunofluorescence positivity of α-fetoprotein (AFP) in the endodermal differentiation, α-smooth muscle actin (SMA) in the mesodermal differentiation, and neuron-specific class III β-tubulin (TUJ1) in the ectodermal differentiation (Fig. 4b). As seen in Fig. 4b, clone #7 from the OA patient 1 stained strongly for the AFP marker. Positivity for this marker was slightly lower in clone #17 from the OA patient 2 but still positive, thus revealing successful endodermal differentiation. Besides, the clones showed successful differentiation into the mesoderm germ layer, as shown by SMA positive staining. Interestingly, spontaneously beating cardiomyocytes were observed in the mesodermal differentiation after 2–3 weeks of induction in all the studied clones. Finally, clone #7 from the OA patient 1 and OA patient 2 clone #17 stained positive for the ectodermal marker TUJ1. These analyses, all together with the previous characterization analyses, revealed the functional pluripotency of the two iPSCs clones, therefore establishing the two iPSC-lines named as MOA1-FiPS4F#7 and MOA2-FiPS4F#17, respectively.

#### Karyotype and DNA fingerprinting analyses

**Figure 5:**
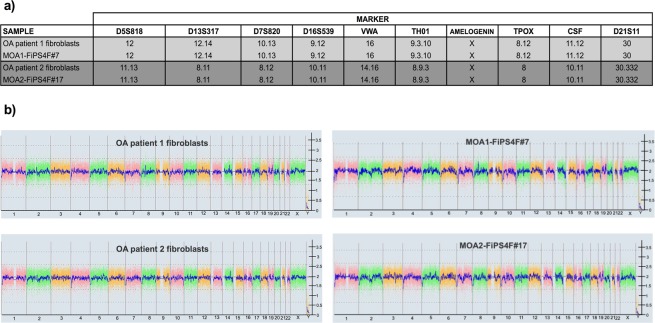
Identity and karyotype analysis of the generated iPSC-lines. (**a**) DNA fingerprinting analysis showing that iPSC-lines MOA1-FiPS4F#7 and MOA2-FiPS4F#17 come from patients’ fibroblasts. The short tandem repeat (STR) locations studied were: D5S818, D13S317, D7S820, D16S539, VWA, TH01, AMELOGENIN, TPOX, CSF and D21S11. (**b**) Whole genome view obtained after the KaryoStat™ analysis of both patient fibroblasts’ and iPSC-lines. The whole genome view displays all somatic and sex chromosomes in one frame with high-level copy number. The smooth signal plot (right y-axis) is the smoothing of the log2 ratios, which depict the signal intensities of probes on the microarray. A value of 2 represents a normal copy number state (CN = 2). A value of 3 represents chromosomal gain (CN = 3). A value of 1 represents a chromosomal loss (CN = 1). The pink, green and yellow colors indicate the raw signal for each individual chromosome probe, while the blue signal represents the normalized probe signal which is used to identify copy number and aberrations (if any).

The derivation of the lines MOA1-FiPS4F#7 and MOA2-FiPS4F#17 from the patients’ fibroblasts was confirmed by DNA fingerprinting analysis (Fig. 5a). Both parental dermal fibroblasts and iPSCs had normal diploid 46, XX karyotype, without acquired detectable abnormalities in the two lines analysed (Fig. 5b).

### Single nucleotide polymorphisms presence within the GDF5, SMAD3, ALDH1A2 and IL1-R1 genes

Results obtained after the analysis of the selected SNPs in the generated iPSC-lines are shown in Table 1. These results correspond to those observed in the parental dermal fibroblasts from which each of the lines was generated, thus showing that the presence of the different alleles in each of the genes remained after reprogramming.

**Table 1:** Summary of the allelic varints detected after single nucleotide polymorphism analysis of the iPSC-lines generated.

			Genotype	
Gene	SNP	At-risk allele	MOA1-FiPS4F#7	MOA2-FiPS4F#17
GDF5	rs143383	T	CT	CT
SMAD3	rs12901499	G	GG	GG
ALDH1A2	rs3204689	C	CC	CT
IL1*-*R1	rs2287047	T	CT	CT

GDF5 (growth differentiation factor 5); SMAD3 (SMAD family member 3); ALDH1A2 (aldehyde dehydrogenase 1 family A2); IL1-R1 (interleukine 1 receptor 1); SNP (single nucleotide polymorphism); MOA1-FiPS4F#7 (iPSC-line from patient with rhizarthrosis and non-erosive hand OA in the right hand); MOA2-FiPS4F#17 (iPSC-line from patient with rhizarthrosis and non-erosive hand OA in both hands).

### Directed chondrogenic differentiation of the iPSCs

**Figure 6:**
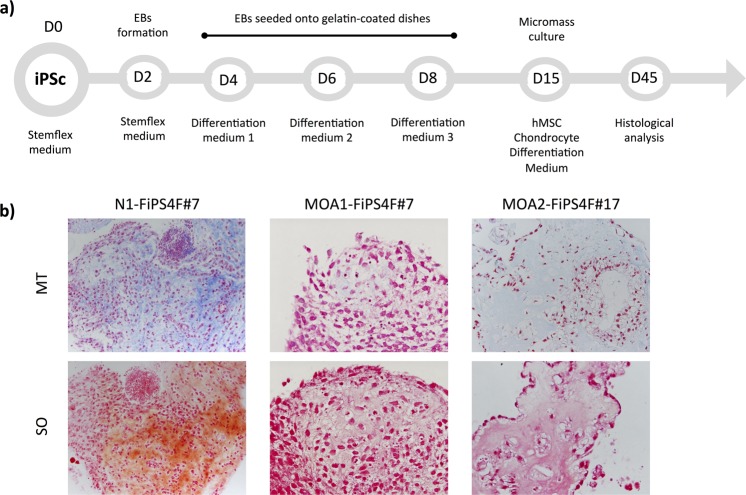
Directed chondrogenic differentiation of the iPSCs. (**a**) General scheme of the differentiation protocol. (**b**) Histological evaluation by means of Masson’s trichromic (MT) and safranin O (SO) staining of the chondrogenic differentiation of the iPSC-line derived from a healthy donor (N1-FiPS4F#7) and the iPSC-lines generated from patients with hand OA (MO1-FiPS4F#7 and MOA2-FiPS4F#17). x20 magnification.

Histological analysis of the micromasses performed after the chondrogenic differentiation protocol (Fig. 6a) showed differences in the levels of collagen (COL) and proteoglycans (PGs) within the matrix produced by the differentiated cells, as seen by Masson’s Trichromic (MT) and Safranin O (SO) sataining (Fig. 6b). The differentiated cells derived from the iPSC-line with no rheumatic diseases (N1-FiPS4F#7, ESi080-A, registered in the Human Pluripotent Stem Cell Registry on December 12, 2019) showed more presence of blue and orange staining, corresponding to COL and PGs content, respectively. In comparison, lower levels of both chondrogenic markers were lower in the micromasses obtained from the iPSC-lines MOA1-FiPS4F#7 and MOA2-FiPS4F#17. High quality chondrogenesis, with chondrocyte-like-rounded cells embedded in an extracellular matrix composed by COL and PGs^7^, were reached to a higher extent in the ‘healthy’ iPSC-line, than in the iPSC-lines derived from the patients with hand OA. These preliminary results highlight the usefulness of the obtained iPSCs lines to further investigate the phenotypes associated to hand OA at cellular level.

## Discussion

We report here for the first time the generation of two iPSC-lines from dermal fibroblasts of patients with rhizarthrosis and non-erosive hand OA (MOA1-FiPS4F#7 and MOA2-FiPS4F#17). Furthermore, both in the iPSC-lines and parental fibroblasts, we studied the presence of sequence variants within genes that have been suggested to be implicated in the pathogeneses of hand OA^17,20–25^, thus establishing the first iPSC cellular models of non-erosive hand OA and rhizarthrosis.

Dermal fibroblasts are an easily accessible cell source with high proliferative capacity, and therefore, a big amount of autologous cells can be obtained from a minimally invasive isolated starting population^26^. That is why we chose to reprogram dermal fibroblasts to generate the iPSC-lines. Contrary to other studies in which harvested cells are reprogrammed immediately after isolation^27,28^, we performed cell characterization and demonstrated the successfully isolation of dermal fibroblasts from skin biopsies of the two donors by using the explant culture technique.

We conducted the reprogramming process by transducing patients’ dermal fibroblasts with Sendai virus vectors carrying the OSKM reprogramming factors. Conversely to the use of retroviral or lentiviral vectors, which can produce insertional mutagenic lesions and chromosomal aberration due to their integration into the host genome^29,30^, Sendai virus is transcribed in the cytoplasm, enabling the clearance of the virus as the cells are passaged^31,32^. In fact, we did detect neither virus presence nor expression of the Sendai virus factors by qRT-PCR in the lines studied, thus showing the generation of two “zero-footprint” iPSC-lines.

It is well known that a major limitation of inducing pluripotency is its low efficiency^33^. In consistency with other studies using Sendai virus vectors in which the efficiency is normally 0.1–1%^34–36^ we obtained a reprogramming efficiency between 0.2–0.95%. Nevertheless, we would like to highlight that the reprogramming efficiency is not a maker of iPSC-line quality since only one high-quality iPSC clone per patient is needed^37^.

When new iPSCs lines are developed, it is extremely important to perform an exhaustive characterization of the new lines, in order to unequivocally define the iPSCs populations, testing functional pluripotency and defining abnormalities that could affect cell behaviour and safety^38^. We have therefore carried out a panel of assays accordingly to the recommendations of the Spanish competent authorities and demonstrated that the established iPSC-lines presented normal karyotype, with no mutations acquired alongside the reprogramming process. The morphology of the iPSC-lines was identical to ESCs and they also presented AP activity, proposed as a reliable pluripotency marker in human ESCs together with the right morphology^39^. iPSC-lines showed ESC-like gene expression profile, and this expression was extended to the protein levels of the key pluripotency factors NANOG, OCT-4, SOX2 and the ESC-specific antigens SSEA-4 and TRA-1–81. At functional level, we demonstrated that both iPSC-lines could differentiate into the three embryonic layers.

Once the generation of iPSCs from patients with OA of the hand has been achieved, a vast of possibilities are opened up for *in vitro* studying the disease. In our case we aimed to deeply characterize the generated iPSC-lines by evaluating the presence of hand OA-associated SNPs in order to gather information about the underlying genetic variants of the cells. We found that the iPSC-lines presented different allelic combinations in the studied genes, therefore enabling researchers with an unlimited cellular tool for studying the role of the specific variants of interest. Furthermore, knowing the genetic variants carried by the cells may simplify the interpretation of future results obtained, for example, after performing chondrogenic differentiation experiments.

Regarding rs143383 variant within the GDF5 gene, it has been proposed that the T allele, both in homozygosis and heterozygosis, associates with lower levels of GDF5 protein^40^, which is activated during development for inducing mensenchymal condensation and subsequent chondrogenic differentiation^21^. Furthermore, it has been shown that GDF5 inhibits catabolic enzymes activities and stimulates the expression of anabolic enzymes in articular cartilage^21,41^, which may explain why this variant is considered a risk factor for OA development. However, GWASs have suggested that association between GDF5 and OA risk is just consistent in populations with knee and, eventually, hip OA^19,40,42^. Although the relationship between this variant and hand OA is still unclear, the establishment of iPSC-lines carrying the polymorphism of interest may help to increase our understanding about the role of this SNP in hand OA pathogenesis.

We also decided to evaluate the presence of the rs12901499 variant within SMAD3 gene, due its essential role as cellular messenger in cartilage integrity maintenance and homeostasis^22^. Several studies have reported that SMAD3 gene rs12901499 polymorphism accelerates chondrocyte maturation^43^, catabolic enzymes production^22^, and increases the risk of osteoarthritis^44^. However, it is still controversial which is the role of this variant in hand OA pathogenesis. The iPSC-lines generated in our study are, to our knowledge, the first iPSC cellular models of hand OA, and they are furthermore carrying a homozygous at-risk allele G. Therefore, we have generated an invaluable cellular tool for helping researchers to precisely elucidate the genetic role of SMAD3 gene rs12901499 polymorphism in the hand OA development.

Both iPSC-lines carried the at-risk C allele within the retinaldehyde dehydrogenase 2 (ALDH1A2) gene, an enzyme implicated in the synthesis of retinoic acid. This finding is biologically relevant since this hormonal signaling molecule is involved in the embryonic development and in adult tissues maintenance^20,45^. Moreover, ALDH activity has been proposed as a marker of chondrocytes from human adult articular cartilage, with enriched production of type II COL^16^. In our study iPSC-lines from patients with rhizarthrosis were generated for the first time. In this sense, Zhu and colleagues observed that patients with severe OA at the base of the thumb had lower levels of the ALDH1A2 enzyme in the cartilage of the affected joint, and that these low levels coincided with the presence of the allele C^24^.

Proinflammatory cytokines are considered important mediators in the osteoarthritic process^25,46^. The pathogenic implication of the interleukin-1 gene family cluster has been reported in hip, knee, or hand OA. Recently, it has been described that coding region and the region harboring the long promoter of the IL1-R1 gene are associated with severe hand OA^47^. Näkki and colleagues analyzed the presence of several SNPs at the level of this gene and observed that the variant rs2287047 was associated with severe bilateral hand OA in the study population^47^. Therefore, we decided to study the presence of this polymorphism in the generated iPSC-lines. In accordance to these results, we observed that the iPSC-lines were heterozygous for the risk allele T. These results make the generated lines a useful tool for *in vitro* evaluating the role of these combinations in the early development and further progression of hand OA.

These iPSC-lines carrying different allelic combinations represent valuable tools for developing molecular studies to elucidate the role of these genes and their related proteins in the pathogenesis of rhizarthrosis and non-erosive hand OA. But since it is the first time that iPSC-lines form patients with hand OA have been generated, it may be still soon to firmly state that it is possible to model the disease via the iPSCs technology. According to Liu and colleagues^48^ the establishment of models of OA standing on the use of iPSC will certainly improve the current knowledge about the pathogenenic processes underlying this disease, therefore shedding light about potential treatments. In this regard, we have performed a preliminary study analysing the *in vitro* chondrogenic differentiation capacity of the iPSC-lines derived from patients with hand OA. As a healthy phenotypic control, we included for analysis an additional iPSC-line that had been previously generated in our group from a donor with no rheumatic diseases^49^. The hypothesis of poor chondrogenic differentiation capacity of the iPSCs generated from hand OA patients was checked against the healthy donor. Excellent chondrogenic differentiation capacity is necessary for modelling early and late OA pathogenesis, as well as cartilage development^37^. Based on previous studies, we have developed a differentiation protocol^37,50^ that mimics the development of pluripotent cells through the primitive streak mesendoderm-mesoderm intermediates–chondrocytes pathway. Additionally, the first step of EBs formation supposedly resembles the early post-implantation embryo and the cells in the EB should therefore be able to differentiate into all cell types^7^. Although more research is needed in order to optimise the current protocols for differentiating iPSCs into chondrocytes^7^, here we demonstrate that the iPSC-lines derived from patients with OA of the hand show less capacity to produce extracellular matrix rich in COL and PGs, after the differentiation process, in comparison to the iPSC-line derived from the healthy donor. These preliminary results are in accordance with previous studies in which the extracellular matrix of chondrocytes derived from patients’ iPSC resembled the one observed during advanced OA^11^.

The pluripotency of the iPSCs makes it possible to differentiate them towards the cell types involved in joint degeneration during OA. In addition to chondrocytes, these iPSCs may be differentiated into muscle cells, synoviocytes or bone cells, thus making it possible to study changes occurring during the disease in all the involved tissues, and thus coinciding with the new consideration of the joint as an organ^51^. We strongly believe that all studies developed to date and the ones that will be conducted hereafter, would proportionate the demonstration that *in vitro* iPSC models can be used to understand the mechanisms implicated in hand OA, to find new therapeutic targets, and to test potential drugs useful for clinical in the near future. Therefore, we hope this research could be the first step towards this goal.

## Methods

### Obtaining and culture of human fibroblasts

This study was approved by the Ethics Committee of Research of A Coruña-Ferrol, Spain (register code 2014/405) and was done in accordance with Spanish laws and regulations regarding the generation of human iPSCs, following a protocol approved by the Spanish competent authorities (*Comisión de Seguimiento y Control de la Donación de Células y Tejidos Humanos del Instituto de Salud Carlos III*). Cell lines will be deposited at the *Banco Nacional de Líneas Celulares* (*BNLC, ISCIII*) following the Spanish legislation. Informed consent was obtained from all donors.

To isolate dermal fibroblasts, 3 mm skin biopsies of two patients (52 and 76-years-old women respectively) with hand OA were obtained by means of a biopsy punch. Skin tissue was cut up into smaller pieces (<1 mm), placed into 6 well dishes (Costar Corning Incorporated) and let them dry at 37 °C until attachment. Once the explants were attached to the plate, culture medium (Dulbecco’s Modified Eagle’s Medium; DMEM; Lonza) supplemented with 10% fetal bovine serum (FBS; Gibco-ThermoFisher Scientific) and 1% penicillin/streptomycin (P/S; Gibco) (10%DMEM) was added carefully, and the plates were incubated at 37 °C in a humidified atmosphere with 5% CO_2_. When cells sprouted from the explants, after one week in culture, the plates were washed with sterile saline solution (Fresenius Kabi) in order to eliminate non-adherent cells and surplus explants, and new 10%DMEM culture medium was added. The culture medium was then replaced every 3 days. When sprouted cells reached 80% confluency, subculturing was performed for cell expansion.

### Fibroblast cultures characterization

Patients’ fibroblasts at the 3rd passage were histologically characterized before reprogramming, and positivity for fibroblast markers was quantified by using the peroxidase/DAB ChemMate^TM^ DAKO EnVision^TM^ detection kit (Dako) following manufacturer’s recommendations. Type I collagen (ab90395, Abcam), FGFR4 (ab44971, Abcam) and vimentin (ab8069, Abcam) primary antibodies were used. Lastly, samples were counterstained with haematoxylin-eosin and visualised in an Olympus BX51M microscope coupled to an Olympus DP70 digital camera (Olympus Iberia S.A.). Pictures were taken employing the CellSens Dimension software (Olympus Iberia S.A.).

### Non-integrative reprogramming of human fibroblasts into iPSCs

**Figure 7:**
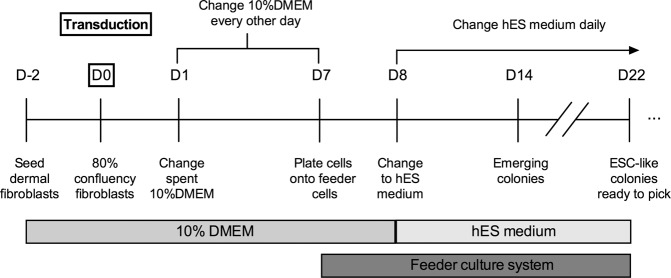
Time course of the followed reprogramming protocol to generate human iPSC-lines.

The reprogramming process was conducted introducing the reprogramming factors Oct4, Sox2, Klf4 and c-Myc (OSKM)^9^ using non-integrative Sendai RNA viruses (CytoTune-iPS Sendai reprogramming Kit, Gibco-ThermoFisher Scientific), following the instructions of the manufacturer, as it is schematized in Fig. 7. Briefly, two days before transduction, 5 × 10^4^ dermal fibroblasts at the 4th passage were plated in 12-well plates (Costar Corning Incorporated) to reach 80% confluency on the day of transduction and cultured in 10%DMEM. Then, 48 hours later, medium was changed for new 10%DMEM medium containing the CytoTune vectors at a 1:1:1:1 ratio. Plates were placed in a 37 °C, 5% CO_2_ incubator overnight. Following the overnight incubation, the spent medium was replaced for fresh 10%DMEM medium every other day for a week. Seven days after transduction the cells were harvested and passaged onto feeder cells (75 Gy-γ-irradiated human foreskin fibroblasts; HFF-1, ATCC). On the next day, spent medium was replaced for human embryonic stem cell (hES) culture medium containing 80% DMEM Knockout without L-glutamine, 20% knockout serum replacement, 1% non-essential aminoacids, 1% Glutamax 100X, 1% P/S, 0.1 mM β-mercaptoethanol and 100 μg/ml basic fibroblast growth factor (bFGF) (all from Gibco). Medium was changed daily. Clonal iPSCs lines were established by manually picking human ESC-like colonies.

### Culture and expansion of human iPSCs

iPSCs were cultured both on feeder layers and on feeder-free layers. In the first case, human iPSCs were seeded onto feeder cells as described above and manually picked once a week. Subsequently, iPSC clones were adapted and cultured in feeder-free conditions on human rh-laminin-521 with Stemflex medium (all from Gibco), following the recommendations of the manufacturer.

### Characterization of the human iPSCs

In this study, several clones were validated by means of colonies morphology, AP activity analysis and gene expression profile, but only one clone from each patient was characterized in detail, as it is described below.

#### Alkaline phosphatase analysis

AP activity was studied in the whole 10-cm Petri dishes (Costar Corning Incorporated) with newly appeared colonies from the two patients using the alkaline phosphatase blue membrane substrate solution kit (AB0300, Sigma-Aldrich Química S.A.) according to the manufacturer’s guidelines. Furthermore, iPSC clones with more than 20 passages were seeded onto a 6 well feeder plate (Costar Corning Incorporated). When colonies appeared, AP activity was also determined. All samples were observed in a Nikon stereomicroscope SMZ745 coupled to a Nikon Digital Slight DS-Fi2 camera (Nikon).

#### RNA extraction and qRT-PCR analyses

5 × 10^5^ dermal fibroblasts at the 4th passage and iPSCs at the 8th-10th passage were harvested and stored as dry pellet at −20 °C until RNA extraction was performed. Also, RNA was extracted from patients’ fibroblasts immediately after reprogramming as a positive control for Sendai virus detection. Total RNA was extracted using the RNeasy Mini Kit (Qiagen) according to the manufacturer’s instructions for animal cell samples. RNA concentrations were measured with NanoDrop^TM^ spectrophotometer (Thermo Fisher Scientific), and the 260/280 and 260/230 nm absorbance ratios were calculated to assess RNA purity. cDNA synthesis was performed from 1 μg of total RNA using the SuperScript^TM^ Vilo^TM^ master mix (Thermo Fisher Scientific), following the instructions of the manufacturer. One μl of the reaction was used to quantify the expression of endogenous pluripotency associated genes (OCT4 ENDO, SOX2 ENDO, KLF4 ENDO, NANOG and CRIPTO) and to assess the silencing of the exogenous reprogramming factor genes and Sendai virus genome. All evaluated genes were analysed in duplicate by qRT-PCR on the LightCycler 480 Instrument (Roche) using LightCycler 480 SYBR Green I Master (Roche). The reactions were conducted using the following parameters: 95 °C for 10 s and 45 cycles at 95 °C for 10 s, 60 °C for 5 s, 72 °C for 10 s. Primers used for the amplification were previously described^36^. Gene expression levels relative to the glyceraldehyde 3-phosphate dehydrogenase (GAPDH) housekeeping gene were calculated by the 2^−ΔΔCt^ method^52^_._

#### *In vitro* EB formation and tri-lineage differentiation

Tri-lineage differentiation was performed through the embryoid body (EB) formation protocol. For EB formation, hanging drop method was used. Briefly, iPSC colonies cultured in feeder-free conditions were trypsinized and dispersed into single cell suspension using TrypLE^TM^ express enzyme 1X (ThermoFisher Scientific). iPSCs were resuspended in hES medium without the pluripotent cytokine bFGF. 20 μl drops with 10^4^ cells were formed on the inner surface of the lid of the culture dish. Then, the lid was carefully inverted and placed on top of the dish, and incubated for 48 hours at 37 °C and 5% CO_2_. After 2 days, the formed EBs were transferred independently to 0.1% gelatin-coated 8 well chamber-slides (Merk), and cultured in differentiation medium (80% DMEM Knockout without L-glutamine, 20% FBS, 1% non-essential aminoacids, 1% Glutamax 100X, 1% P/S and 0.1 mM β-mercaptoethanol) to stimulate endodermal differentiation. For mesodermal differentiation, EBs were maintained in differentiation medium supplemented with 100 μM ascorbic acid (Sigma-Aldrich Química S.A.). To stimulate ectodermal differentiation, EBs were independently transferred to matrigel-coated 8 well chamber-slides (Merk) and cultured in a specific differentiation medium containing DMEM F-12, 1% P/S, 1% Glutamax 100X, 0,5% N2 supplement (Gibco) and 1% B27 supplement (Gibco). In all cases medium was changed every other day during 3 weeks.

#### Immunofluorescence analyses

Immunofluorescence analyses of undifferentiated human iPSCs and cells sprouted from EBs during trilineage differentiation were performed. For this purpose, cells were fixed 10 min ant room temperature (RT) with 4% paraformaldehyde (Sigma-Aldrich Química S.A.) and washed twice with phosphate buffered saline (PBS; Dako). When necessary, cells were permeabilized with 0,1% Triton-X-100 (Sigma-Aldrich Química S.A.) in PBS at RT for 10 min and blocked with Triton block solution containing 0,75% glycine (Sigma-Aldrich Química S.A.), 2% bovine serum albumin (Sigma-Aldrich Química S.A.) and 0,1% Triton-X-100 in 0,01 M PBS pH 7,4 for 15 min. Primary antibodies diluted in Triton block solution were added and incubated over night at 4 °C. Thereafter, cells were washed with PBS and secondary antibodies in Triton block were added. One-hour incubation in the dark was performed. Slides were washed with PBS, counterstained with DAPI (Sigma-Aldrich Química S.A.) and coverslipped using fluorescent mounting medium (Dako). All the samples were visualized in an A1R confocal scanning microscope (Nikon).

Primary antibodies used were as follows: NANOG (ab109250, Abcam. 1:100), OCT-4 (sc-5279, Santa Cruz Biotechnology. 1:50), SOX2 (sc-365823, Santa Cruz Biotechnology. 1:500), SSEA-4 (sc-21704, Santa Cruz Biotechnology. 1:500), TRA1-81 (ab16289, Abcam. 1:100), α-fetoprotein (AFP) (ab 133617, Abcam. 1:500), α-smooth muscle actin (SMA) (ab 7817, Abcam. 1:100) and neuron-specific class III β-tubulin (TUJ1) (T8660, Sigma-Aldrich Química S.A. 1:500). Secondary antibodies used were goat anti-rabbit-PE (sc-3739, SantaCruz. 1:200) and rabbit anti-mouse-FITC (F0313, Dako. 1:200).

#### Kariotype analysis

Karyotype analysis of patients’ fibroblasts at the 5th passage and finally selected iPSCs clones with more than thirty passages in feeder-free culture was conducted by ThermoFisher Scientific using the KaryoStat^TM^ service (ThermoFisher Scientific). The KaryoStat™ assay allows for digital visualization of chromosome aberrations with a resolution similar to g-banding karyotyping.

#### Autentication and mycoplasma testing

To confirm lines identity STR analysis was performed. Genomic DNA was extracted from iPSCs and patients’ fibroblasts at the 4th passage using the DNeasy Blood and Tissue Kit (Qiagen), and sent to the genomic service at the “Alberto Sols” Institute of Biomedical Research (*Madrid*, Spain) for analysis. Absence of mycoplasma contamination in the iPSCs was checked by PCR.

### Single nucleotide polymorphism analysis

For SNP analysis, DNA was extracted from both donors’ fibroblasts at the 4th passage and iPSCs clones using the DNeasy Blood and Tissue Kit (Qiagen) according to the manufacturer’s instructions, on the QIAcube automated system (Qiagen). Presence of SNPs within the genes GDF5 (variant rs143383), SMAD3 (variant rs12901499), ALDH1A2 (variant rs3204689) and IL1-R1 (variant rs2287047) were studied by Sanger sequencing using the primers listed in Table 2.

**Table 2:** Table showing the primer sets used to assess the presence of single nucleotide polymorphisms within the genes GDF5 (variant rs143383), SMAD3 (rs12901499), IL1-R1 (variant rs2287047) AND A2BP1 (variant rs716508).

Gene	SNP	Primer Forward (5′-3′)	Primer Reverse (5′-3′)	Size
GDF5	rs143383	caggcctgtgagtgtgtgtg	cagcagtagcagcagaagga	376 bp
SMAD3	rs12901499	ttaaagcaggggagtggcac	aagcacaggcccccaaatta	368 bp
ALDH1A2	rs3204689	ctcttccaaggagatgtcagc	acacacacaccccaaaactg	332 bp
IL1-R1	rs2287047	accagcctccagagaagaaa	gtgcatagctgactttggatgt	411 bp

GDF5 (growth differentiation factor 5); SMAD3 (SMAD family member 3); ALDH1A2 (aldehyde dehydrogenase 1 family A2); IL1-R1 (interleukine 1 receptor 1); SNP (single nucleotide polymorphism); bp (base pair).

### Directed chondrogenic differentiation

Subsequent differentiation of the iPSCs into relevant cell types to study the diseases is needed in order to use them as cellular *in vitro* models of hand OA. Therefore, we developed a chondrogenic differentiation protocol based on previous protocols^35,50^ with several modifications. For this differentiation experiment, the iPSC-lines MOA1-FiPS4F#7 and MOA2-FiPS4F#17 were employed, as well as another iPSC-line as a control (N1-FiPS4F#7), which was previously generated by our group from a donor with no rheumatic diseases^49^. The chondrogenic differentiation protocol consisted in the formation of EBs, sequential addition of specific growth factors and culture of the cells in three-dimensional pellets or micromasses. Thus, iPSCs at the 70–100th passage were disrupted and detached from the feeder layer using a ‘stripper’ micropipette (Origio MidAtlanticDevices) and a flexed capilar (Gynétics). Pieces of colonies were transferred to a non-treated 60 mm culture dish (Corning) and cultured in suspension for 48 hours in Stemflex medium (ThermoFisher) in order to form spontaneous EBs. After 48 hours, EBs were seeded onto gelatin-coated dishes and culture medium was replaced for differentiation medium containing DMEM F-12, 1% P/S, 1% Insulin transferrin selenium (ITS; Gibco), 1% FBS, 10 ng/ml Wingless-type family member 3a (Wnt3a; R&D Systems), and 10 ng/ml human activin A (StemCell Technologies) (differentiation medium 1). After two days, differentiation medium 1 was changed for a new differentiation medium containing DMEM F-12, 1% P/S, 1% ITS, 1% FBS, 10 ng/ml bone morphogenic protein-2 (BMP-2, R&D Systems), and 10 ng/ml GDF-5 (R&D Systems) (differentiation medium 2). Then, 48 h later, differentiation medium 2 was replaced by new differentiation medium composed by DMEM F-12, 1% P/S, 1% ITS, 1% FBS, 10 ng/ml bone morphogenic protein-2 (BMP-2, Gibco), 10 ng/ml GDF-5, 50 μg/ml Ascorbic Acid (Sigma), and 10 ng/ml transforming growth factor-beta 3 (TGF-b3, Prospec-Tany Technogene Ltd) (differentiation medium 3). For further differentiation and matrix deposition, cells were cultured on hMSC Chondrocyte Differentiation Medium (Lonza) supplemented with 10 ng/ul TGF-b3 for several days until cells stopped proliferating and then, cells were trypsinized and transferred to propylene tubes for micromass formation. Micromass pellets were cultured in hMSC Chondrocyte Differentiation Medium supplemented with 10 ng/ul TGF-b3 for 30 days, with media changes every 3–4 days. After 30 days, cell aggregates were fixed in paraformaldehyde, paraffin-embedded and stained with MT and SO for visualization of typical cartilage extracellular matrix proteins.
